# The prevalence and clinical seriousness of medication discrepancies identified upon hospital admission of pediatric patients

**DOI:** 10.1186/s12913-018-3795-1

**Published:** 2018-12-14

**Authors:** Rana Abu Farha, Khawla Abu Hammour, Sayida Al-Jamei, Raja AlQudah, Mohammed Zawiah

**Affiliations:** 1 Department of Clinical Pharmacy and Therapeutics, Faculty of Pharmacy, Applied Science Private University, Amman, Jordan; 2 Department of Biopharmaceutics and Clinical Pharmacy, Faculty of Pharmacy, The University of Jordan, Amman, Jordan; 3 Department of Pharmacy Practice, Faculty of Clinical Pharmacy, Al-Hodeida University, Al-Hodeida, Yemen

**Keywords:** Prevalence, Discrepancies, Pediatrics, Medication reconciliation, Jordan

## Abstract

**Background:**

Medication discrepancies are seen frequently in hospital setting upon admission or discharge. Medication Reconciliation service is a practice designed to ensure that patients’ medications are ordered in a correct manner upon hospital admission, thus reducing the risk of having medication discrepancies. This study aimed to determine the prevalence of medication discrepancies and their clinical seriousness in pediatric patients at the time of hospital admission.

**Methods:**

A prevalence cross-sectional study was conducted at the pediatric departement at the Jordan University hospital between March–May 2018. During the study period, 100 pediatric patients were enrolled using a convenience sampling method. Patients’ medical records were reviewed by two clinical pharmacist-reserachers to obtain patients' demographic, medical, and admission medication information. All parents were interviewed to obtain information regarding their children’s Best Possible Medication History (BPMH). Following data collection, differences between patient’s current admission medications and the BPMH were identified as medication discrepancies, and then they were classified into either undocumented intentional or unintentional discrepancies.

**Results:**

Among the 100 medication records reviewed, 13.0% (13 out of 100) contained at least one unintentional discrepancy, with the majority (*n* = 11, 84.6%) being classified to be associated with mild potential harm to patients. Of those discrepancies, 8 were omission of medications (61.5%) and 5 were addition of unnecessary medication (38.5%). On the other hand, 35.0% (35 out of 100) of medication records contained at least one intentional undocumented discrepancy.

**Conclusions:**

This study revealed that unintentional medication discrepancies exist at the time of hospital admission for pediatric patients but with low proportion. The low proportion of medication discrepancies might be explained by the recent implementation of medication reconciliation service at the studied hospital. Also, intentional undocumented discrepancies were common, which may carry a potential harm to such vulnerable population at discharge. These data may inform the need for a strict policies to regulate medication documentation, thus decreasing the possibilities of medication errors.

## Background

When patients are admitted to hospitals, a list of all medications, doses, routes, and frequencies of administration is obtained by different healthcare providers [1]. Obtaining incomplete medication history is attributed to a quarter of hospital prescribing errors [1–3]. In heathcare setting, these errors are associated with increasing the incidence of adverse effect affecting patients’ quality of life [4]. In addition, almost 60% of medication errors occur at admission, transfer, or discharge from the hospital as reported by Rozich and Roger [5]. Furthermore, 60% of the identified medication errors were found to cause harm in adult [6].

In the pediatrics healthcare setting, medication errors are still an alarming problem, with the prevalence of medication errors was found to be three time higher than that identified in adults [7]. Pediatrics are considered a vulnerable population to medication errors [8]. The unique vulnerability of pediatric population is a consequence of several aspects, including the need of weight-based dosing, the need to perform an extemporaneous dispensing or compounding of medications, the need to dilute medications from stock solutions, the lack of mature kidney or liver that might affect medications’ metabolism or elimination, the dependent of such population on their caregiver to administer their medication and their inability to report any experienced adverse effects [9].

Among the medication errors that might affect pediatrics are unintentional discrepancies identified during transition of care [10]. To reduce medication discrepancies either at admission [2] or at discharge [11], medication reconciliation is needed. The process of reconciliation will identify any possible medications’ discrepancies including medication addition, duplications, omission, or any inappropriateness of current medications, thus, ensuring an accurate and complete medication list upon hospital admission or discharge [1, 10]. By doing this, medication errors will be reduced thereafter [2]. Accordingly, as a requirement for hospital accreditation, the Joint Commission International (JCI) has mandated the implementation of medication reconciliation for the purpose of reducing medication discreapncies and medication errors [12, 13].

Till now, only few studies have investigated the prevalence and nature of medication discrepancies in pediatric populations [2, 7, 11, 14, 15]. Thus, this study is the first of its kind in Jordan to identify the prevalence of medication discrepancies during hospital admission for a pediatric population. The study was conducted at Jordan University Hospital, which is the first hospital in Jordan received the JCI accreditation and implemented medication reconcilitaion service. Thus, this study may reflect the impact of the implementation of medication reconciliation service on the prevalnce of medication discrepancies.

## Methods

### Study design, subjects and clinical setting

This is a prevalence cross-sectional study that was conducted on a cohort of pediatric patients admitted to pediatrics department at Jordan University hospital, Amman- Jordan. Jordan University Hospital is considered as the biggest and first teaching hospital in Jordan that was established in 1975. It has received the JCI accreditation in April 2017, after which the hospital started to apply several standards as a requirement to improve quality of health care provided to patients. Among these standards is the introduction of medication reconciliation service.

The study was conducted between the 21th of March and the 16th of May 2018. During the study period, 230 pediatric patients were approached using a convenience sampling method to assess their eligibility for inclusion in this study. Inclusion criteria included: child younger than 18 years of age, receiving at least one chronic medication (regular prescription) prior to admission, having one of his/her parents accompanying him/her and expected to stay more than 24 h in the hospital.

### Data collection

Firstly, before data collection, two clinical pharmacist-researchers holding a master degree in clinical pharmacy were trained on how to implement medication reconciliation service. They received two hours training session on how to collect the Best Possible Medication History (BPMH) and how to identify medication discrepancies. The trainer was a PhD holder in pharmacy who have a good experience in medication reconciliation research.

Once data collection was started, the pharmacist-researchers assessed patient’s eligibility for inclusion by reviewing patient’s medical file. Once patients were found to be eligible for inclusion, the nature and the purpose of the study were explained to their parents and a parental written informed consent was obtained for each patient. In case of child 9 years and older an additional child assent was obtained [16].

Patients’ information was collected using a structured data collection form that was adopted from a previous study [17]. The data collection form included two main sections: 1) patient’s demographic information (age and gender), 2) and medical information (patients’ medical problems, prior to admission medications, admission medication list, and intended length of stay in hospital). Information was collected by the two clinical pharmacist-researchers through reviewing patients’ medical records.

Following medical record review, the clinical pharmacist-researchers interviewed parents as another source to obtain patient's BPMH lists. Parents were asked to verify the accuracy of information obtained from the medical records and to fill in the gap with any omitted medication that was not recorded. All types of medications including prescription, over the counter medications and herbal supplements were requested from all parents.

### Assessment of medication discrepancies

In this study, medication discrepancy was defined as any difference, either intentional or unintentional, between patient’s current admission medications and the BPMH. Identification of medication discrepancies was performed by the two clinical pharmacist-researchers. After identifying discrepancies, pharmacist-researchers reviewed and discussed all identified discrepancies with the responsible prescriber, and a clinical judgment was made by the pharmacist-researchers to classify discrepancies into undocumented intentional or unintentional discrepancies.

An undocumented intentional discrepancy is defined as the discrepancy occurring when the prescriber has made an intentional choice to change, add or discontinue a medication but without documenting this change within the medical record. While, an unintentional discrepancy is occurring when the prescriber unintentionally changed, added or omitted a medication the patient was taking prior to admission. Unintentional discrepancies are considered medication errors that need to be prevented or resolved.

Unintentional discrepancies fall into several categories: omission of a required drug previously used, addition of a drug not previously used and not justified by the patient’s clinical condition, duplication of drugs, dosage discrepancies, frequency discrepancies, administration route discrepancies, or dosage form discrepancies. Also, they were classified by their potential to cause harm into three categories based on the classification used by Cornish et al. [18]: 1) discrepancies with the potential to cause mild discomfort or clinical deterioration; 2) discrepancies with the potential to cause moderate discomfort or clinical deterioration, and 3) discrepancies with the potential to result in severe discomfort or clinical deterioration.

### Sample size calculation

The following formula was used to calculate the minimal sample size for inclusion in the study:

$$ \mathrm{n}\kern0.5em =\kern0.5em \mathrm{P}\kern0.5em \times \kern0.5em \left(1\hbox{-} \mathrm{P}\right)\kern0.5em \times \kern0.5em {\mathrm{z}}^2/{\mathrm{d}}^2 $$

Where P″ is the prevalence of medication discrepancies, where 22% of pediatrics patients were found to have at least one unintentional discrepancy in a previous study conducted in Toronto, Canada [2]. While “d” is the desired precision (10%), and “z” is equal to 1.96 corresponding to 95% level of confidence.

Based on this formula, a minimum sample size of 66 patients was considered representative for the purpose of this study.

### Ethical consideration

The study was commenced after obtaining the ethical approval by the Institutional Review Board at the Jordan University Hospital (Reference number: 65/2017). Ethical standards defined by the World Medical Association Declaration of Helsinki guideline were followed while conducting this study [19]. Patients’ confidentiality were well-preserved by using pseudonymous data collection forms.

### Statistical analysis

Statistical Package for Social Science (SPSS) version 24.0 (IBM Corporation, Armonk, NY, USA) was used to conduct statistical analyses. The descriptive statistics were used to analyze quantitative and qualitative variables using mean/standard deviation (SD) and frequency/percentages, respectively.

## Results

### Demographic and medical characteristics of the study participants and their parents

**Fig. 1:**
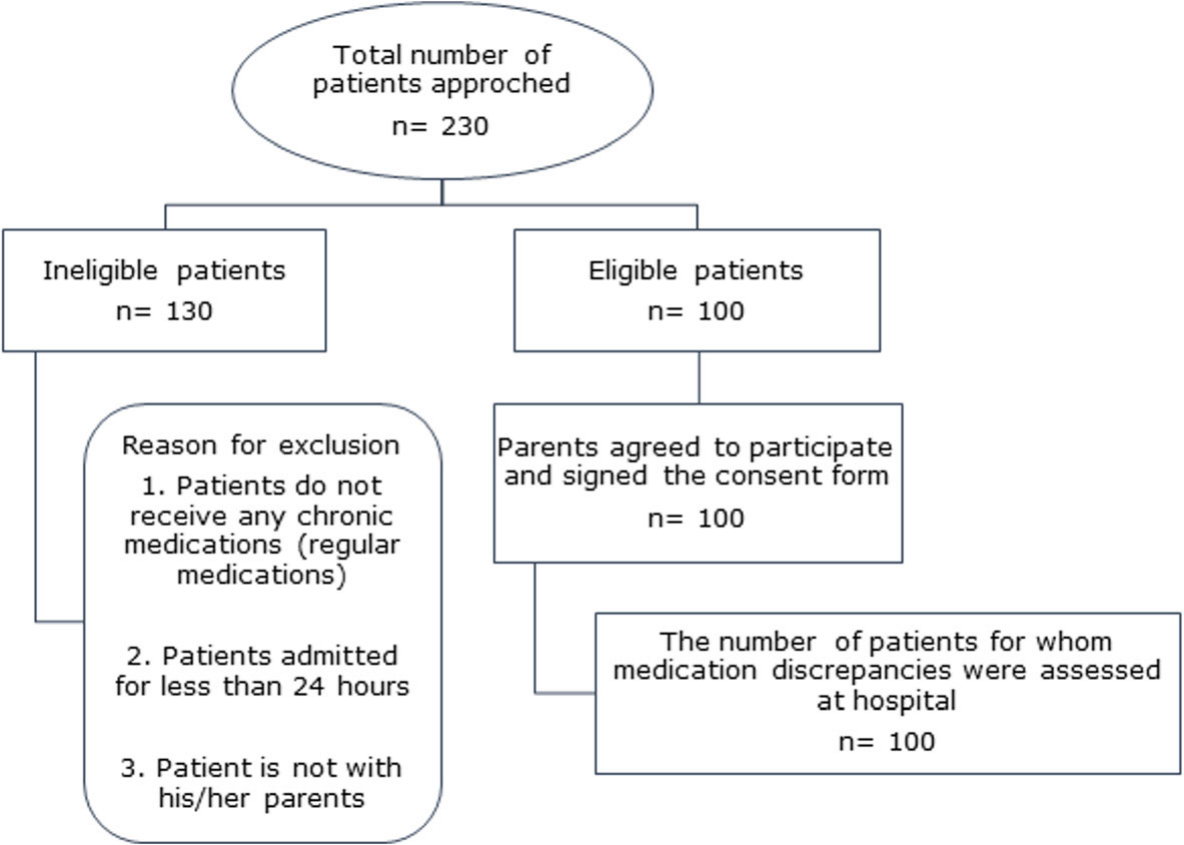
Patient enrollment in the study

During the study period, 230 pediatric patients were screened for eligibility criteria. Of those, 100 patients were eligible for inclusion. Parents of all eligible patients agreed to participate in the study and signed the informed written consent (response rate 100.0%) (Fig. 1).

Mean age of children included was 6.6 years (SD ± 4.2); with 61 (61.0%) being males. Among the 100 medication records reviewed, a total of 411 medications were identified, with a mean of 4.1 (SD ± 2.6) admission medications per patient prescribed upon hospital admission. All patients’ BPMH was obtained by reviewing the medical record and interviewing their parents (*n* = 100, 100.0%). The average number of pre-admission medications was 2.6 (SD ± 1.7), and the average number of medical conditions was 1.4 (SD ± 0.7). Epilepsies were the most commonly encountered medical conditions (*n* = 25, 25.0%) followed by Asthma (*n* = 13, 13.0%). Patients had an average of 5.0 days (SD ± 2.3) length of stay at hospital (Table 1).

**Table 1:** Socio-demographic and medical characteristics of the study sample (*n* = 100)

Parameter	Mean (SD)	*n* (%)
Child Age, years	6.6 (4.2), (0.2–17)	
Child Gender		
Males		61 (61.0)
Female		39 (39.0)
Number of Pre-admission Medications	2.6 (1.7)	
Number of Admission Medications	4.1 (2.6)	
Number of Medical Conditions	1.4 (0.7)	
Length of Stay (days)	5.0 (2.3)	
Pediatric sub-specialty		
Nephrology/Urology		13 (14.0)
Neurology		23 (7.5)
Respiratory		15 (13.5)
Endocrinology		9 (31.0)
Gastroenterology		7 (2.0)
Oncology/hematology		2 (15.5)
Infectious		27 (10.0)
Rheumatology		3 (6.0)
Ophtalmology		1 (0.5)
Most Common Medical Conditions		
Epilepsy		25 (25.0)
Asthma		13 (13.0)
Diabetes mellitus type 1		8 (8.0)

### Medications discrepancies among study participants

**Fig. 2:**
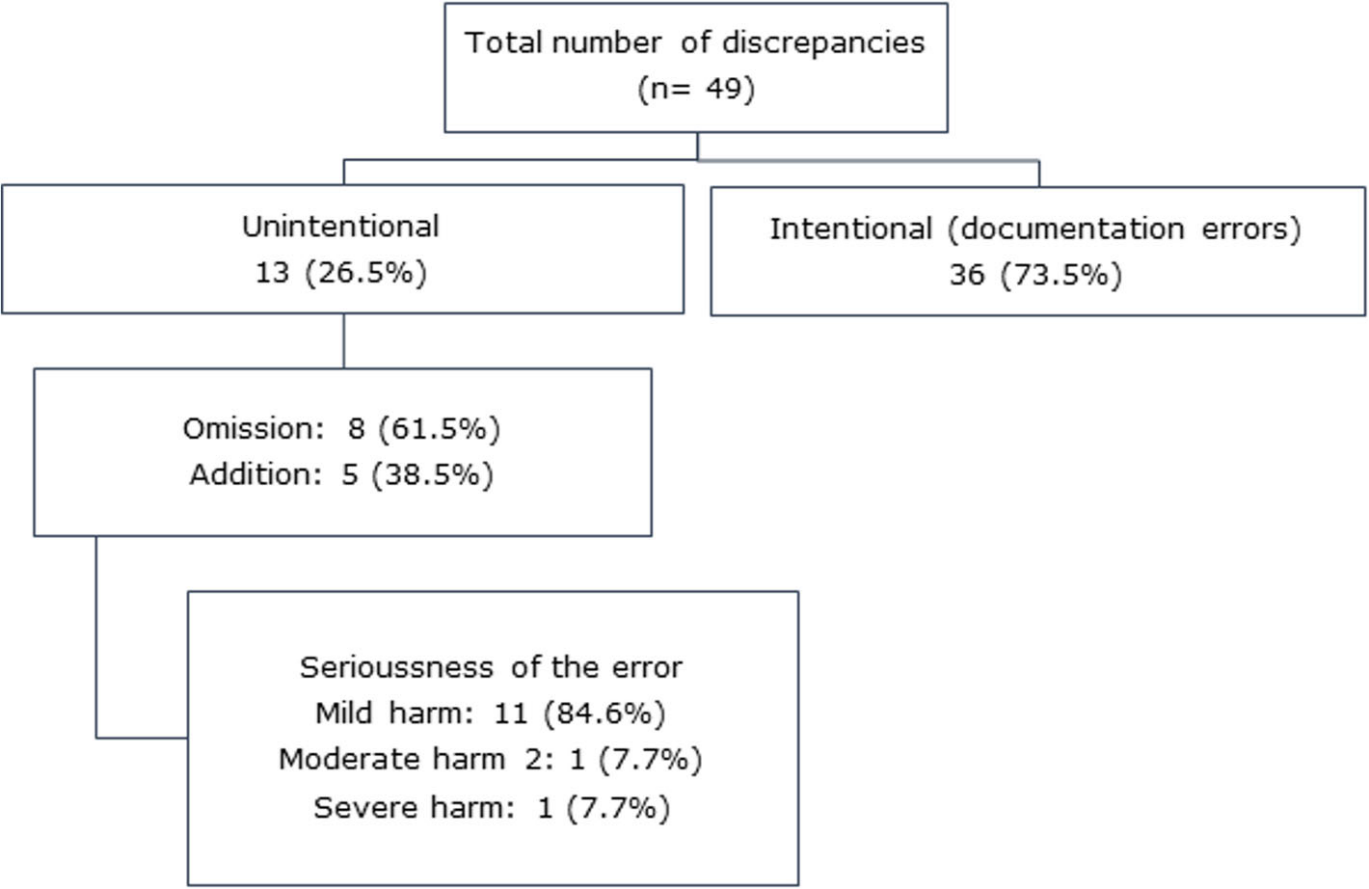
Classifications of medication discrepancies identified among study participants (*n* = 100)

Among the 411 medication identified, 49 (11.9%) medications showed a discrepancies with the BPMH. Among those discrepancies, 36 discrepancies (73.5%) were justified by the responsible physician, which indicates a documentation discrepancies (documentation errors). The remaining 13 discrepancies (26.5%) were identified as unintentional discrepancies (medication errors). Of those discrepancies, 8 were omission of medications (61.5%) and 5 were addition of unnecessary medications (38.5%) (Fig. 2). Overall, 13.0% (13 out of 100) of medication records reviewed contained at least one unintentional discrepancy, while 35.0% (35 out of 100) contained at least one intentional discrepancy.

**Fig. 3:**
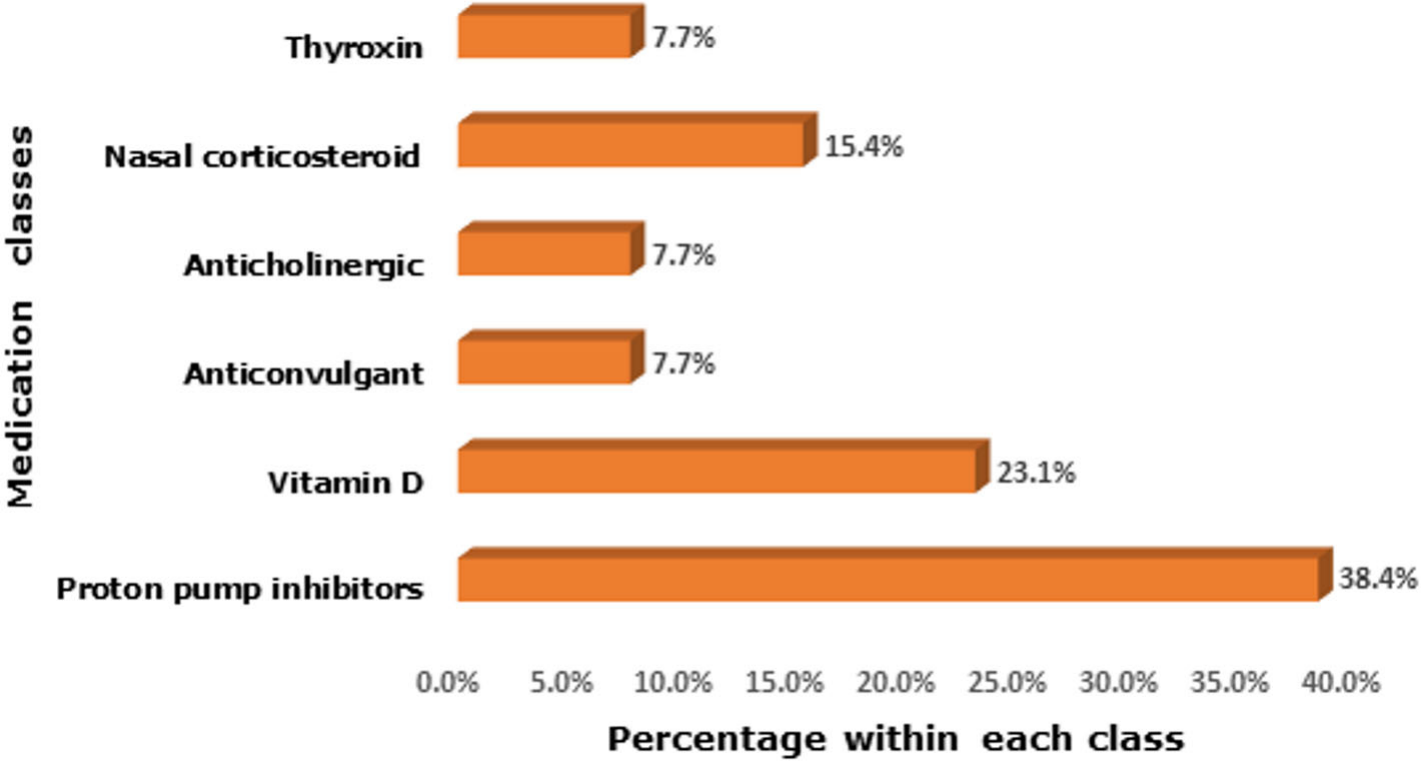
Types of drug classes/therapies associated with unintentional medication discrepancies

Proton pump inhibitors were the most commonly drug therapies involved in unintentional medication discrepancies (*n* = 5, 38.4%), followed by vitamin D (*n* = 3, 23.1%), Fig. 3. The majority of identified unintentional discrepancies (*n* = 11, 84.6%) were classified to be associated with a mild potential harm or deterioration to patients.

## Discussion

Medication errors have been estimated to be associated with significant morbidity and mortality worldwide and were found to cause 7000 patients deaths annually in the United States [20]. Accordingly, several safety ensuring services including medication reconciliation were mandated by the JCI to ensure patient safety in hospital setting, and is considered now as an essential requirements for hospital accreditation in many countries [12]. Most of the studies regarding the prevalence of medication discrepancies were conducted on adult populations [6, 21, 22] and few were conducted on the perdiatric population [2, 7, 11, 14, 15]. Thus, this study aimed to identify the prevalence of medication discrepancies during hospital admission for pediatric patients.

In this study, BPMHs for all patients were obtained by reviewing medical records and interviewing parents. Similar resources were reported by previous study by Coffey et al., where parental recall and medication vials/home medication list were the most frequent used resources to obtain information about BPMH [2]. Other study conducted in Canada stated that patient interview was the most complete resource for medication information, followed by community pharmacy records [23].

Among the medication records reviewed in this study, only 13% contained at least one unintentional discrepancy, with the majority being associated with a mild potential harm to patients. This proportion of medication errors was lower than that identified in adults in a previous study conducted at the internal medicine department at Jordan University hospital, where 47% of patients’ medical records found to have at least one unintentional discrepancy [21]. Similar low proportion of errors was obtained in a previous study on pediatrics, where only 22% of pediatrics patients found to have at least one unintentional discrepancy, of these, 71% were of low clinical importance [2]. Another study conducted in the Mott Children’s Hospital in Ann Arbor, Michigan reported almost similar findings where 26% (18 of 69) of the charts reviewed contained at least one discrepant medication [11].

This low proportion of errors in our study may be justified by the fact that Jordan university hospital had received the JCI accreditation before conducting this research. This might put a pressure on all healthcare providers to comply with accreditation requirements. Patients' medication histories were collected twice at each hospital admission in most cases; first at the emergency department, followed by prescribers’ verification at the pediatrics departments. Also, we were surprised by the role of nurses who adopted the medication reconciliation form to check for any possible medication discrepancies, which create a difference on the completeness and accurateness of patients’ medication records. Also, the pediatrics department at Jordan University hospital is the only department within the hospital containing a clinical pharmacist working to provide clinical pharmacy service to prevent/resolve any possible medication errors. This mean that the collaborative work between all healthcare providers at this department might be the main contributors for such low rate of medication errors.

Despite the low proportion of the unintentional discrepancies identified in this study, still 35% of medication records contained at least one intentional discrepancy. Those intentional discrepancies occur when physician intended to make a change of patients’ home medication upon admission, but without documenting this change. This could potentially lead to accidental changes of medication orders at discharge, which may carry significant potential harm for patients [24]. Coffey et al. found almost similar findings where 30% of pediatric patients had at least one undocumented intentional discrepancy upon hospital admission [2]. Findings of this study clearly demonstrate the urgent need of policies and procedures that regulate medication documentation within the medical records, thus having a complete and accurate medication reconciliation process.

It is remarkable that the most frequent medications involved in the unintentional discrepancies were proton pump inhibitors (5 out of 13), which were added without therapeutic justification. The overuse of proton pump inhibitor was highlighted previously in several studies conducted in Jordan [25], as well as overseas [26, 27]. Also, proton pump inhibitors were found to be the main medications involved in drug discrepancies in previous research conducted at the internal medicine department at Jordan University hospital [21]. This finding draw the attention of the need of certain policies that regulate the prescription of certain classes of overused medications.

It is worth mentioning that this study was conducted in a single center (the pediatrics department at Jordan University hospital) which could somewhat limits its generalizability. Furthermore, the study relied on a subjective evaluation by the researchers to determine the seriousness of medication discrepancies which could not be a true measure of patient harm. Finally, the identified rate of medication discrepancies in this study may underestimate the actual rate of medication discrepancies on other hospitals due to the fact that Jordan University hospital started to implement medication reconciliation service before conducting this study.

## Conclusion

This study revealed that unintentional medication discrepancies exist at the time of hospital admission for pediatric patients at a tertiary teaching hospital in Jordan. The proportion of such discrepancies was low, and this might be explained by the recent implementation of medication reconciliation service at the studied hospital. Intentional undocumented discrepancies were common in this study, which may carry a potential harm to such vulnerable population at discharge. These data may inform the need for a strict policies to regulate medication documentation, thus decreasing the possibilities of medication errors. A future multicenter observational study is needed to investigate the actual rate of medication discrepancies among pediatric patients and to evaluate the seriousness of those discrepancies on patients' health.

## Abbreviations

BPMH: Best Possible Medication History
SPSS: Statistical Package for Social Science
